# Chronic N-acetylcysteine treatment improves anhedonia and cognition in a mouse model of the schizophrenia prodrome

**DOI:** 10.3389/fnbeh.2022.1002223

**Published:** 2022-09-26

**Authors:** Lukas Marius Bühner, Sampath K. T. Kapanaiah, Dennis Kätzel

**Affiliations:** Institute of Applied Physiology, Ulm University, Ulm, Germany

**Keywords:** N-acetylcysteine, cyclin-D2, early-intervention, prodrome, schizophrenia, cognitive flexibility, anhedonia, working memory

## Abstract

Schizophrenia is a severe psychiatric disorder whose neurodevelopmental pathogenesis includes a prodromal phase before its diagnostically decisive—namely psychotic—symptoms are present. This prodrome is characterized by cognitive and affective deficits, and it may constitute a critical time period for an early therapeutic intervention to improve or even prevent further disease development. N-acetylcysteine (NAC) is an easily repurposable compound that has recently shown promise in improving non-psychotic symptoms in patients with established schizophrenia. Its therapeutic mechanism may involve the amelioration of circuit abnormalities like a hyper-glutamatergic state and oxidative stress in cortex which have been proposed to drive the pathogenesis of this disease. However, it is currently unknown to what extent NAC can actually improve prodromal aberrations. To investigate this preclinically, we deployed the cyclin-D2 knockout mouse model (CD2-KO) that shares physiological and behavioral abnormalities with the schizophrenia prodrome, including a hyperactive CA1 region, and cognitive and affective deficits. Applying NAC chronically in drinking water (0.9 g/l) during development (∼P22–P70), we found that excessive novelty-induced hyperlocomotion was neither ameliorated during (∼P68) nor after (∼P75) treatment; similarly, T-maze working memory (tested after treatment; ∼P84) was unaffected. However, once chronic NAC treatment was resumed (at approximately P134) in those mice that had received it before, working memory, cognitive flexibility (tested under NAC), and anhedonia (sucrose-preference, tested 1 day after NAC-treatment stopped) were improved in CD2-KO mice. This suggests that chronic NAC treatment may be a therapeutic strategy to improve some cognitive and affective dysfunctions in the schizophrenia prodrome.

## Introduction

Schizophrenia is a severely debilitating disorder with a suspected lifetime prevalence of 0.7% and an average age of onset between 16 and 30 years (Owen et al., 2016). This disorder is characterized by positive (or psychotic), cognitive and negative symptoms. While the positive symptoms like hallucinations and delusions may be relatively well controlled in about 65% of patients, no effective and approved treatments yet exist for the other two symptom domains which may manifest as anhedonia, social withdrawal, lack of motivation or impairments of working memory, attention and cognitive flexibility. Such symptoms amount to a major part of the disease burden as they significantly impair daily-life functioning (Owen et al., 2016). The search for effective treatment options has included the off-label clinical investigation of food supplements and drugs already approved for other diseases that can relatively easily be re-purposed for treating schizophrenia. Among investigated anti-oxidant, anti-inflammatory and anti-glutamatergic compounds, N-acetylcysteine (NAC) treatment has consistently shown effective improvements in the negative and cognitive symptom domains according to recent meta-analyses (Zheng et al., 2018; Çakici et al., 2019; Firth et al., 2019; Yolland et al., 2019). NAC may act through multiple, synergistic mechanisms including the increase of glutamate import into glia cells via the cystine/glutamate antiporter (Baker et al., 2008; Durieux et al., 2015; Wright et al., 2016; McQueen et al., 2018), through the elevation of extra-synaptic glutamate activating preferentially inhibitory presynaptic mGlu2/3 receptors (Conn and Pin, 1997; Baker et al., 2008; Zavodnick and Ali, 2014; Wright et al., 2016), and through its anti-oxidant effect, as a precursor of glutathione, protecting peri-neuronal nets and thereby parvalbumin-positive inhibitory interneurons (das Neves Duarte et al., 2012; Cabungcal et al., 2013, 2014; Kätzel et al., 2020).

In recent years, it has been repeatedly suggested that *early interventions*—i.e., therapies that are applied already in the prodromal stage that precedes the onset of psychosis and, hence, the diagnosis of schizophrenia—may be the most effective treatment of this disorder (McGorry, 2011; Moghaddam, 2013; Sommer et al., 2016). Negative and cognitive symptoms already present in the prodrome and their treatment could not only be beneficial directly, but may also prevent progression of the disease and possibly the onset of psychosis. Currently it is unknown, to our knowledge, if NAC treatment could be an effective early intervention. Therefore, we utilized the cyclin-D2 knockout mouse model that displays certain endophenotypes and behavioral deficits that are reminiscent of the schizophrenia prodrome (Grimm et al., 2018). Like prodromal patients (Schobel et al., 2013), this mouse displays hyperactivity of the CA1-region of the hippocampus, but not of the subiculum (Gilani et al., 2014). At the behavioral level, it displays murine correlates of all three symptom domains (Grimm et al., 2018): (i) deficits of rule-reversal and rule-shift learning (i.e., cognitive flexibility) and of working memory assessed by rewarded alternation in the T-maze in the cognitive domain; (ii) anhedonia assessed by sucrose-preference testing in the negative domain; and (iii) elevated novelty-induced hyperlocomotion, that is partially resistant against anti-dopaminergic treatment in the positive domain. Hence, this mouse model represents a useful tool to investigate pharmacological treatments of psychological impairments occurring early in schizophrenia; therefore, we assessed if chronic NAC treatment –starting at juvenile age to mimic an early intervention—was effective in ameliorating such deficits.

## Methods

### Subjects and treatment

**FIGURE 1:**
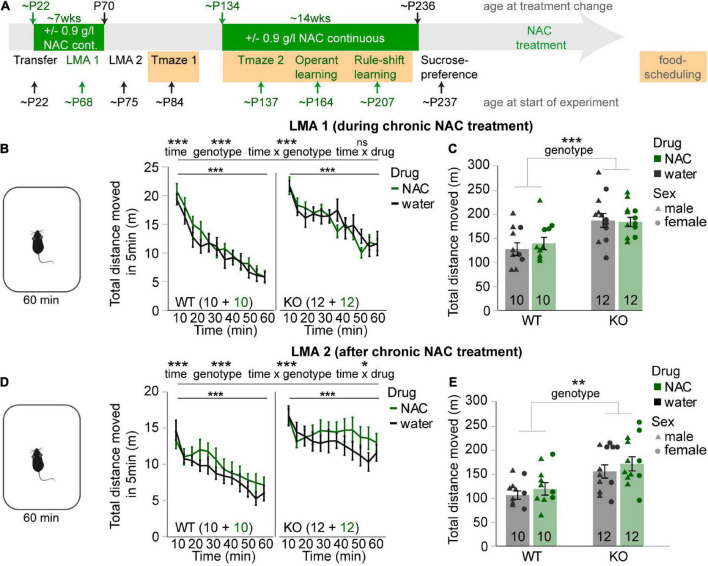
N-acetylcysteine treatment does not affect novelty-induced hyperlocomotion in cyclin-D2 knockout mice. **(A)** Timeline of episodes of chronic NAC-treatment (green bars) and behavioral testing relative to the age of mice; episodes of food-deprivation are highlighted in orange. Tests that were done *with* NAC-treatment on the same day are in green font, tests *without* simultaneous NAC-treatment in black. Note that the second NAC-treatment was started 3 days before testing in the second T-maze experiments started and was stopped on the evening before the sucrose-preference test started. **(B,D)** Average distance moved over 60 min in the first **(B)** and second **(D)** novelty-induced locomotor activity (LMA) test displayed in 5 min bins for NAC-treated (green) and untreated (black) mice of the genotype indicated in the bottom left of each subpanel, also displaying n-numbers. Main effects and interactions from overall RM-ANOVA are indicated above panels; main effect of interval for RM-ANOVA within each subgroup indicated directly above data. No main effect of drug was found in either RM-ANOVA. **(C,E)** Same data and color-code as displayed in neighboring panels **(B,D)** but distance moved was summed up across time to allow univariate ANOVA, and data of individual animals is shown in addition, coded by treatment and sex; only a significant main effect of genotype was found. *p < 0.05; **p < 0.01; ***p < 0.001. Error bars, S.E.M.

All experiments were performed in accordance to the German Animal Rights Law (Tierschutzgesetz) 2013 and were approved by the Federal Ethical Review Committee (Regierungspräsidium Tübingen) of Baden-Württemberg, Germany (license numbers TV1505). In total, a mixed-sex cohort of 44 Cyclin-D2 knockout (CD2-KO) and wild-type littermates (WT) was bred from heterozygous parents for this study and were genotyped before weaning with a previously published protocol (Grimm et al., 2018). Upon weaning (P21), mice were brought from a central holding facility to an experimental holding room that was integrated into the laboratory where behavioral testing took place. Upon arrival, mice were assigned cage-wise to the treatment with NAC or vehicle so as to achieve an even distribution of treatment conditions across mice of both genotypes and sexes (counter-balancing across litter-mates was not possible because all siblings of one sex were kept in one cage throughout the study and therefore received the same treatment via their drinking water); 12 KO (5 females) and 10 WT mice (3 females) received NAC, whereas 12 KO (6 females) and 10 WT mice (3 females) received vehicle only. Mixed-sex group-sizes were determined based on prior data collected on the T-maze rewarded alternation task and the rule-shift assay (Grimm et al., 2018), data from both sexes was pooled for all analysis as the number of females was too low to investigate sex-specific differences. NAC (Sigma-Aldrich, DE, Cat# A7250) was applied chronically in drinking water at a concentration of 0.9 g/l, in accordance with earlier studies (see Table 1) from P22 or P23 onward until P70. After an intermittent application of normal drinking water to investigate potential therapeutic effects *after* juvenile treatment, NAC application was re-started at approximately P134 in the same mice that had received NAC initially and was continued without further interruption until 1 day before sucrose-preference testing started at approximately P236 (see timeline in Figure 1A). Mice were housed in groups of 2–5 in individually ventilated Type-II-Long cages (Green Line, Tecniplast, IT), enriched with sawdust, sizzle-nest™, and cardboard houses (Datesand, GB), and maintained at a 13 h light/11 h dark cycle with the light phase starting at 7 AM.

**TABLE 1:** N-acetylcysteine-induced therapeutic effects in psychiatrically relevant rodent models.

1a. Schizophrenia-related rat models							

Model			NAC application	Cognitive domain	Negative domain	Other changes	References
BSO			Chronic p.o. 10 and 30 mg/kg/d P68–89	↑OM (NOR)	↑ Social interaction	↑ Efficacy of aripiprazole ↑ BDNF	Rogóż et al., 2022
SIR			Chronic i.p. 150 mg/kg/d P63–77	↑OM (NOR)	↑Social interaction	↑Rearing, ↑ PPI ↑ Clozapine efficacy ↓ proInflam. cytokines ↓ KYN/KYN-A ↓ DA (STR); ↑DA (FC)	Möller et al., 2013
NVHL			Chronic 0.9 g/l d-water P5–50	–	–	↑ PVI and PNNs ↓ Oxidative stress ↑MMN, PPI	Cabungcal et al., 2014
MIA + MA			Chronic s.c. 150 mg/kg/d P51–64	↑OM (NOR)	↑Social interaction	↑ PPI ↑ NA (FC, STR) ↓ Oxidative stress	Swanepoel et al., 2018
Poly-I:C and PUS			Chronic i.p. 220 mg/kg/d P30–59	↑WM (alternation)	↑Social interaction	↑ PPI (♀ only) ↓ LMA ↓ Oxidative stress ↑ PVI (♀ only)	Monte et al., 2020
APO-SUS			Chronic, 0.9 g/l d-water P5–40, 2 g/l P40–90	↑Cognitive flexibility	–	↓ Oxidative stress ↑ myelination	Maas et al., 2021
MAM			Chronic 0.9 g/l d-water P11–25	–	–	↑ PVI and PNNs ↓ Oxidative stress	Zhu et al., 2021
MAM			Chronic i.p. 250 mg/kg/d P75–90	–	↑Social interaction	↓ MK801-induced LMA	Lopes-Rocha et al., 2022
MAM			Acute i.p. 150–500 mg/kg	–	↑Social interaction	↓ MK801-induced LMA	Lopes-Rocha et al., 2022
PCP			Acute i.p. 90 mg/kg	↑WM (alternation)	↑Social interaction	↓ Glutamate release	Baker et al., 2008
MA			Acute i.p. 100 and 300 mg/kg	–	–	↓ MA-induced LMA	Fukami et al., 2004

** 1b. Schizophrenia-related mouse models**							

**Model**			**NAC application**	**Cognitive domain**	**Negative domain**	**Other changes**	**References**

G72Tg			Chronic 1 g/l d-water 3–8 weeks	↑SLTM (MWM)	–	–	Otte et al., 2011
ketamine			Chronic s.c. 10 mg/kg P5–21; 1 g/l d-water from P22; or P35–56	↑WM (alternation) ↑Cognitive flexibility ↑OM (NOR)	↑Social interaction ↑ Social novelty pref.	↑ PPI ↑ PVI ↓ Oxidative stress	Phensy et al., 2017a,b
*Gclm* KO			Chronic 0.9 g/l d-water P20–35	–	–	↓ MMP9/RAGE ↑ PVI and PNNs	Dwir et al., 2021
-/KYN			7 days i.p. 100 mg/kg/d	↑SLTM (food location)	–	↓ KYN/KYN-A ↓ Oxidative stress	Blanco Ayala et al., 2021
*Grm5* KO			Acute i.p. 50 and 100 mg/kg	–	–	↑ PPI	Chen et al., 2010
Amph			Acute i.p. 60 mg/kg	–	–	↓ Amphetamine sensitization-induced LMA	Herrmann et al., 2017

**1c. Models related to other brain disorders**							

**Primary disorder**	**Species**	**Model**	**NAC application**	**Cognitive domain**	**Negative domain**	**Other changes**	**References**

MDD	Rat	CLI	Chronic i.p. 100 mg/kg/d, P76–89	↑WM (alternation)	↓Depression ↓Anhedonia (SPT)	↑ HC volume ↑ HC monoamines ↓ Anxiety	Chakraborty et al., 2020b
	Rat	CLI	Chronic i.p. 100 mg/kg/d, P76–89	–	↓Anhedonia (SPT)	↓ Anxiety ↓ Adrenal and spleen volume ↓ Corticosterone	Chakraborty et al., 2020a
	Rat	OBX	10 days i.p. 50 and 100 mg/kg/d	–	↓Depression (FST)	↑ HC and FC SOD activity	Smaga et al., 2012
	Rat	CUMS	1 week p.o 50 and 100 mg/kg/d	–	↓Depression (FST) ↓Anhedonia (SPT)	↑ HC serotonin ↓ proInflam cytokines	Fernandes and Gupta, 2019
	Rat	Alcohol abstinence	3 days i.p.50 and 100 mg/kg/d	–	↓Depression (FST, TST)	↓ GRIN2A/B ↑ HC serotonin	Yawalkar et al., 2018
	Rat	-	Acute i.p. 15–150 mg/kg	–	↓Depression (FST)	–	Ferreira et al., 2008
	Rat	OBX	Acute i.p. 25–100 mg/kg	–	–	↓ Cocaine-seeking	Frankowska et al., 2014
	Mouse	Repeated audible noise	Chronic p.o. 325 mg/kg/d for 30 days	–	↓Depression (TST) ↓anhedonia (SPT)	↓Anxiety ↑ Exploration ↓ Oxidative stress	Mahmoodzadeh et al., 2021
	Mouse	–	Acute i.p. 25 mg/kg	–	↓Depression (TST)	↑Efficacy of imipramine and escitalopram	Costa-Campos et al., 2013
OCD	Mouse	–	Acute i.p. 150 mg/kg	–	–	↓Marble burying	Egashira et al., 2012
Aging	Mouse	–	Chronic 3 g/kg food, for 10 weeks	↑SLTM (MWM)	–	–	Ikonne et al., 2019
AD	Mouse	ApoE4	Chronic^a^, 5 g/kg food	↑WM (alternation)	–	↓ Aggression	Shea, 2007
	Mouse	SAMP8	Chronic s.c. 100 mg/kg/d for 4 weeks	↑ Conditioned avoidance	–	–	Farr et al., 2003
HD	Mouse	R6/1	Chronic i.p. 500 mg/kg/d for 8–12/17 weeks	–	↓Depression (FST)	↑ Motor function ↑ Mitochondrial function ↓ GluN2B ↑ Glutamate	(Wright et al., 2015, 2016)
	Mouse	R6/1	Acute i.p. 500 mg/kg	–	↓Depression (FST)	–	Wright et al., 2016
AE	Rat	WAG/Rij	Chronic p.o. 500 mg/kg/d for 30 days	↑OM (NOR)	↓Depression (FST)	Epilepsy worsens! ↑ mGluR2	Tallarico et al., 2022

^a^Applied in combination with ALCAR (acetyl-L-carnitine; 1 g/kg diet) and SAM (S-adenosyl methionine; 100 mg/kg diet). AD, Alzheimer’s disease; AE, absence epilepsy; Amph, repeated amphetamine application (amphetamine sensitization); APO-SUS, apomorphine-susceptible rat; BSO, L-buthionine-(S,R)-sulfoximine developmental model of SCZ; CLI, neonatal/juvenile clomipramine application model of depression; CUMS, chronic unpredictable mild stress model of depression; DA, dopamine; d-water, drinking water; FC, frontal cortex; FST, force-swim test; Gclm, glutamate-cysteine ligase modulatory subunit; HC, hippocampus; HD, Huntington’s Disease; KYN/KYN-A, kynurenine/kynurenic acid; LMA, locomotor activity; MA, methamphetamine developmental model of SCZ; MAM, methylazoxymethanol acetate developmental model of SCZ; MDD, major depressive disorder; MMP9/RAGE, matrix-metalloprotease 9/receptor for advanced glycation end-products; MIA, developmental LPS-based maternal immune activation model of SCZ; MMN, mismatch-negativity of the auditory evoked potential; MWM, Morris water-maze; NA, noradrenaline; NOR, novel-object recognition test; NVHL, neonatal ventral hippocampal lesion model of SCZ; OBX, bulbectomization model of depression; OCD, obsessive compulsive disorder; OM, short-term object memory; PCP, phencyclidine model of SCZ; PNNs, perineuronal-nets; Poly-I:C, polyinosinic:polycytidylic acid viral mimetic developmental model of SCZ; PPI, pre-pulse inhibition of the startle response; PUS, peripubertal unpredictable stress; PVI, parvalbumin-positive interneurons; SIR, social isolation rearing; SLTM, spatial long-term memory; SOD, superoxide dismutase (anti-oxidant mechanism); SPT, sucrose-preference test; TST, tail-suspension test; WM, working memory.

### Behavioral assessment

Behavioral testing was conducted as previously described (Grimm et al., 2018) during the light phase between 9 AM and 6 PM by experimenters blind to the group identity of mice during experimentation. Where the same test was repeated, each mouse was tested at the same time of day upon each repetition.

#### Novelty-induced locomotor activity

Locomotor activity was measured in a novel open field (OF), which was a clear Type-III plastic cage (425 × 266 × 185 mm; Tecniplast, DE) filled with clean sawdust. Movements were recorded for 60 min using CCTV cameras (Sentient, UK) installed centrally above the open-field cages. The video-recordings were fed into a single image frame through a CCTV-system (Dahua Inc., CN), digitized through an A/D converter (The Imaging Source, DE), and processed by ANY-maze (San Diego Instruments, US) to extract the distance moved in 5 min time intervals. The first LMA testing was done around P68, i.e., during the last days of the initial chronic NAC treatment; the second LMA test was conducted at P75/P76, i.e., 5–6 days after the treatment had ceased.

#### T-maze rewarded alternation assay of working memory

Spatial working memory (SWM) was assessed in a T-shaped maze (W 10 cm, L 40 cm; H 10 cm) with a red PVC floor, transparent Perspex walls, and metal food wells at the end of each goal arm. A first sequence of T-maze testing were conducted within 20 days after the first NAC treatment had ceased at P70 (starting at P84 on average). Before testing, mice were food-restricted to maintain ca. 85–90% of their free-feeding body weight, accustomed to the condensed milk reward (10% fat, diluted 1:2 in drinking water; REWE, DE) in their home cage, and habituated to the maze and reward first with their cage mates and then individually. After 1–3 days of habituation (depending on how well the mice explored the maze), a series of 10 test sessions with 10 trials each was applied over 5-6 days, running mostly two sessions per day; in some cases, the first two sessions were conducted on two consecutive days as mice were still running slowly. Each trial consisted of a sample phase where the mice could collect the reward in one pseudo-randomly assigned goal arm while the other one was blocked off, a delay during which the mice were maintained on the experimenters arm, and a test phase during which both goal arms were accessible and the previously unvisited one had to be entered to obtain another reward. The delay was varied between 5 s in the first six sessions, 1 s in sessions 7 and 8, and 20 s in the last two sessions. SWM-performance was determined as %correct choices out of the total number of trials per session and this indicator was averaged between session 5 and 6, 7 and 8, and 9 and 10 to obtain values for the delays 5, 1, and 20 s, respectively. Sessions 7 and 8 were conducted in a massed paradigm with 20–25 s inter-trial interval (Bygrave et al., 2016; Grimm et al., 2018), whereas all other sessions were conducted as round-robin with an inter-trial interval of 2–4 min. The T-maze experiment was repeated starting 3 days after NAC treatment was resumed (i.e., with concomitant chronic NAC treatment). In this second sequence of 10 sessions, some mice ran more slowly, especially in later trials, so that sessions consisted of 6–10 trials, rather than 10 trials consistently; one KO mouse of the water-only group did not contribute data in the second sequence as it did not consume the milk reward.

#### Operant rule-shift learning

Mice were trained and tested in custom-made 5-choice operant boxes described previously (Kapanaiah et al., 2021) in 45 min long sessions conducted once per day. Mice were food-restricted again, as for the prior SWM-testing, accustomed to the strawberry milk reward (Müllermilch, DE) in their home-cage and the operant box, and then trained to obtain a 40 μl milk reward for poking into any of the 5 holes of the 5-choice wall of the box, which were all illuminated until poking. Once mice had obtained at least 40 rewards in one 45 min session—but not earlier than session 4—they began training in an operant 2-choice task (*forward learning*). Here, either hole 2 or 4 of the 5-choice wall were illuminated for up to 8 s in each trial at equal probability across trials, and poking into the illuminated hole (*correct response*) was followed by reward delivery (20 μl) at the receptacle on the opposite side of the box. Two seconds after exit from the reward receptacle, a new trial started with the illumination of a choice hole. Poking into non-illuminated holes (*incorrect response*) or failing to poke within the stimulus duration of 8 s or the subsequent limited hold time of 1 s (*omission*) lead to a 3 s time-out (instead of reward) during which the house-light was switched off, before a new trial started. Once a mouse had performed at least 16 sessions and had achieved a response accuracy [number of correct/(number of correct + incorrect responses)] of ≥70% in two consecutive sessions, the rule according to which reward could be obtained was switched from a stimulus-related to a spatial rule (*rule-shift*); i.e., in the rule-shift period, the poking of one of the two holes (2 or 4, assigned counterbalanced across the cohort) was always rewarded, irrespective of its illumination. Therefore, in 50% of trials the rewarded hole was also illuminated (congruence of old and new rule) whereas in the remainder the rewarded hole was not illuminated but the other (distractor) hole was (conflict of old and new rule). The capacity for rule-shifting was assessed in the latter trials according to the number of incorrect responses into the lit distractor hole (*incorrect lit* responses, *perseverative* errors), the number of correct responses into the unlit hole (*correct unlit* responses according to new rule only), their *accuracy unlit* ratio [*correct unlit*/(*correct unlit* + *incorrect lit*)], and the responses into the holes 1, 3, and 5 that were wrong choices according to both rules (*never reinforced* or *random errors*). Additionally, the number of *correct lit* and *incorrect unlit* responses were recorded for the trials where the two rules were not in conflict to monitor the adherence to the successful rule when applicable. Mice were trained for 10 sessions, by which time all mice had achieved the learning criterion of an *accuracy unlit* of ≥70% in two consecutive sessions. Three mice were excluded before the experiment due to health issues unrelated to treatment or genotype. Two further mice were excluded after training had started: one WT mouse receiving water did not learn the forward rule within 55 sessions, one KO mouse receiving NAC experienced a failure of the operant box during reversal learning which perturbed its learning curve (but was included for the analysis of forward learning).

#### Sucrose-preference testing of anhedonia

The second chronic NAC treatment was terminated on the evening before the test day, and around 9 AM of the test day, animals were single-housed in cages with two water bottles filled with normal drinking water. Around 6 PM, one bottle was refilled with a solution of 1% sucrose in drinking water and both bottles were present throughout the night. Bottles were weighed again between 8 and 9 AM. According to the weights of the bottles at all-time points, the liquid consumption was determined and sucrose-preference was calculated as the ratio of consumed sucrose solution to consumed total liquid intake. Three mice were excluded before the experiment due to health issues unrelated to treatment or genotype.

### Statistical analysis

Statistical analyses were performed using SPSS 26.0 (IBM, US). Two-way repeated-measures analyses of variance (two-way RM-ANOVA) were used in experiments that included data from multiple training or test days with drug and genotype as between-subject factors. Similarly, univariate ANOVAs were used on normally distributed data with a simple between-subjects design. In both cases, significant effects in the ANOVA were further investigated using simple main-effects pairwise *post-hoc* tests with Šidák adjustments for multiple comparisons. The Mann-Whitney-*U* (MWU) test was used where the normality of the data was not assumed. A *p* < 0.05 was considered statistically significant (*), and *p* < 0.1 are reported as trend (#). All data are presented as mean values ± standard error (S.E.M.).

## Results

### N-acetylcysteine treatment does not affect novelty-induced hyperlocomotion in cyclin-D2 knockout mice

Novelty-induced LMA was measured over 60 min first during (∼P68) and then after (∼P75) chronic NAC treatment (Figure 1A). In both cases, interval data showed a clear spatial habituation over time and novelty-induced hyperlocomotion in CD2-KO mice (*p* < 0.001, main effects of time, genotype and of time-genotype interaction; RM-ANOVA; Figures 1B,D). This was confirmed by the aggregated distance moved in 60 min (*p* < 0.01, effect of genotype, univariate ANOVA; Figures 1C,E). NAC application did not rescue such hyperactivity at either time point nor did it affect total movement in general (*p* > 0.1 for drug-genotype-time interaction, RM-ANOVA; Figures 1B,D; *p* > 0.1 for effect of drug and drug-genotype interaction, univariate ANOVA, Figures 1C,E). To the contrary, during the second testing, there was a time-drug interaction (*p* > 0.05, RM-ANOVA) driven by a slower decrease of LMA over time that was qualitatively apparent in the NAC-groups of both genotypes (Figures 1B,D). In summary, long-term NAC-treatment during juvenile and adolescent age did not appear to normalize this correlate of the positive symptom domain in CD2-KO mice.

### Concomitant chronic N-acetylcysteine treatment mildly improves working memory across subgroups

**FIGURE 2:**
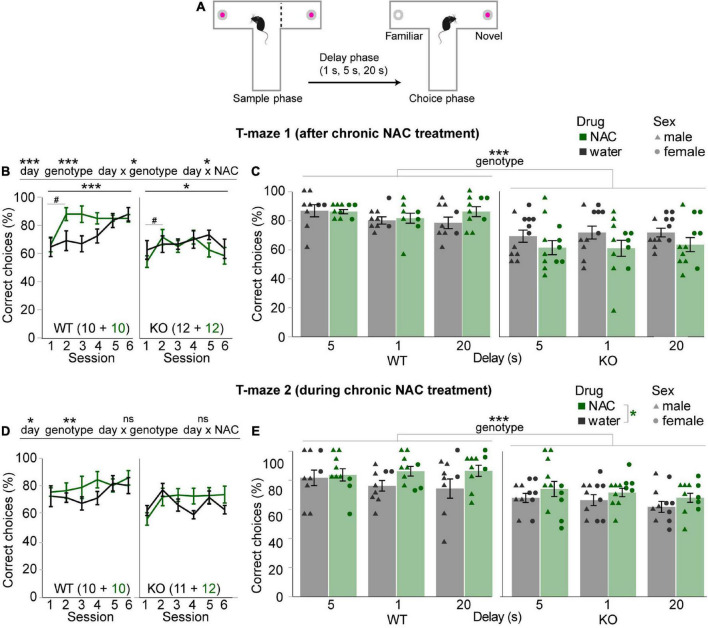
Mild improvement of spatial working memory by concomitant chronic N-acetylcysteine treatment. **(A)** Design of the T-maze rewarded alternation task. **(B,D)** Average SWM accuracy (%correct choices) in the first six training sessions (all run with a 5 s delay) of the first **(B)** and second **(D)** T-maze experiments shown for NAC-treated (green) and untreated (black) mice of the genotype indicated in the bottom left of each subpanel, also displaying n-numbers. Main effects and interactions from overall RM-ANOVA are indicated above panels; main effect of day for RM-ANOVA within each subgroup indicated underneath where significant; pairwise Šidák *post-hoc* tests were conducted between consecutive sessions within subgroups due to significant day-genotype and day-NAC interactions in panel **(B)** and indicated trends of improvement in NAC-subgroups (#) between the first two sessions. **(C,E)** Data from the same experiments as displayed in neighboring panels **(B,D)** with the same color code, but showing averages between the two last sessions with the same delay (stated on *x*-axes) and data of individual animals, coded by treatment and sex. Main effects of overall RM-ANOVA across delays and subgroups are indicated, if significant, above panels (genotype; **C,E**) and in the legend (NAC-treatment; **E**). No significant interactions were found. ^#^*p* < 0.1; *p < 0.05; **p < 0.01; ***p < 0.001. Error bars, S.E.M.

Subsequently, mice were tested in a T-maze rewarded alternation assay of spatial working memory (SWM; Figure 2A) for 10 sessions without resuming NAC-treatment. During the first six sessions of training, there was a significant improvement of performance (*p* < 0.001, effect of day, RM-ANOVA) that was marginally stronger from session 1 to session 2 in the mice that had received prior NAC-treatment (*p* < 0.05 for day-drug interaction, RM-ANOVAs across all six and across the first two sessions, *p* < 0.1 for within-subject Šidák *post-hoc* comparison across the first 2 days within each of the subgroups; Figure 2B), hinting toward a mildly beneficial long-term effect of the NAC treatment. However, this benefit was not maintained and could not ameliorate the clear SWM-deficit in the CD2-KO mice (*p* < 0.001, effect of genotype, Figure 2B). This assessment was confirmed when analyzing the last six sessions of the schedule presented as two-session averages within each of the delays (5, 1, and 20 s, in order of testing). Across delays, KO-mice performed significantly worse than WT mice (*p* < 0.001), while there was no effect of NAC-treatment or drug-genotype interaction (*p* > 0.05, RM-ANOVA; Figure 2C).

Due to this unclear result, chronic NAC-treatment was resumed in those animals that had received NAC during adolescence, and the test schedule was repeated. While, in this case, the significantly lower performance of CD2-KO mice during the first six training days and the different delay conditions was maintained (*p* < 0.01, effect of genotype, RM-ANOVAs) there was also a small, but—across delay challenges—consistently higher SWM performance in animals receiving NAC compared to those receiving just normal drinking water across both genotypes (*p* = 0.027 for effect of drug, *p* = 0.717 for drug-genotype interaction; RM-ANOVA; Figures 2D,E). These results suggest that chronic NAC treatment might have a mildly cognition-enhancing effect during the treatment, especially when cognitive demands are changing due to delay challenges. The number of trials performed per session was not significantly different between subgroups (*p* > 0.1, effects of group, drug, and drug-genotype interaction, RM-ANOVA).

### Chronic N-acetylcysteine treatment improves cognitive flexibility in cyclin-D2 knockout mice

**FIGURE 3:**
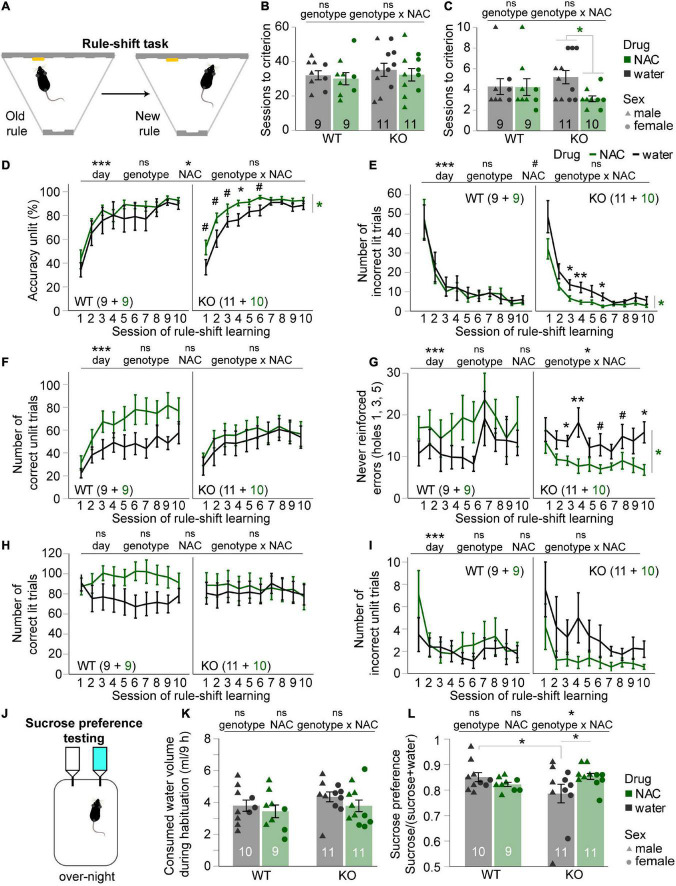
Chronic N-acetylcysteine treatment improves cognitive flexibility and sucrose preference (anhedonia) in cyclin-D2 knockout mice. **(A)** Illustration of the first, cue-related and the second, spatial rule animals had to acquire in the operant rule-shift task. **(B,C)** Number of sessions needed after the rule-shift to achieve the training criterion of ≥70% *accuracy* **(B)** or *accuracy unlit* **(C)** in two consecutive sessions; data of individual animals is shown in addition, coded by treatment and sex. Results of MWU-tests between treatments groups within genotype are indicated where they were significant. Genotype-related main effects of univariate ANOVA are shown above each panel. **(D–I)** Averages of the performance parameters indicated on the y-axes in the 10 rule-shift learning sessions shown for NAC-treated (green) and untreated (black) mice of the genotype indicated in the bottom left or top right of each subpanel, also displaying n-numbers. Main effects and genotype-NAC interactions from overall RM-ANOVA across the first six sessions (where a performance improvement can be assumed) are indicated above panels. Significant effects of drug treatment from RM-ANOVAs within each genotype are indicated on the right of each sub-panel where significant; for these cases, significant pairwise *post-hoc* comparisons within each session are indicated directly above the data. **(J)** Illustration of task. **(K)** Consumed volume of liquid during the habituation session, approx. 12 h after NAC treatment had ceased and immediately before sucrose-preference testing was started. **(L)** Sucrose preference score achieved by each subgroup indicated on the *x*-axis (genotype) and by color (NAC, green; water, black); data of individual animals is shown in addition, coded by treatment and sex. Significant drug-genotype interaction found in overall univariate ANOVA is indicated on top (no significant main effects of drug or genotype were found). Šidák *post-hoc* comparisons are indicated directly above data, where significant. *p < 0.05. Error bars, S.E.M. ^#^*p* < 0.1; *p < 0.05; ***p < 0.001. Error bars, S.E.M.

To explore this further, we assessed another cognitive domain, namely cognitive flexibility, using an operant rule-shift learning assay in which we have previously found a deficit in CD2-KO mice (Grimm et al., 2018). Animals had to switch from an acquired cue-guided to a new spatial rule in order to obtain a reward (Figure 3A). The number of sessions needed to acquire the forward, cue-guided rule at the pre-set criterion of ≥70% accuracy on two consecutive days was not affected by drug or genotype (*p* > 0.5, pairwise MWU-tests between subgroups; Figure 3B). The number of sessions needed to achieve a criterion of ≥70% accuracy in the decisions according to the new rule (*accuracy unlit*) in two consecutive sessions was analyzed as a first indicator of rule-shifting capacity. In contrast to our previous study (Grimm et al., 2018), however, we did not find that CD2-KO mice needed significantly more sessions to achieve criterion, even when just regarding the non-treated groups alone (*p* = 0.263, MWU-test within untreated group, Figure 1C; note however, that the learning appeared much faster in the current study in general, which is likely related to the fact that mice had been trained for multiple months according to the cue-guided rule before rule-switching in our previous, but not in the present study). Importantly, NAC-treated CD2-KO mice learned significantly faster than untreated CD2-KO mice (*p* = 0.014, MWU-test), which was not the case in the WT-group (*p* = 0.849, MWU-test, Figure 1C). We therefore analyzed selectively which factors drove the improved performance in NAC-treated knockouts investigating accuracy and several error types across the individual sessions. The main performance criterion *accuracy unlit*, which increased steadily across the first six sessions in all groups and faster in NAC-treated mice (*p* < 0.001 for effect of day, *p* = 0.046 for effect of NAC, *p* > 0.4 for effect of genotype; RM-ANOVA) was higher in NAC-treated compared to untreated CD2-KO mice (*p* = 0.015 for effect of genotype, RM-ANOVA within KO-group; Figure 3D). Notably, the beneficial effect of NAC in KO-mice was mainly due to the reduction of both *perseverative* and *never reinforced* errors (*p* < 0.05, effect of drug, RM-ANOVA within the KO group across the first six sessions, Figures 3E,G), whereas the number of correct responses was not affected (*p* > 0.5, RM-ANOVA, Figure 3F). Likewise, there was no difference in the number of correct or incorrect responses in the trials where old and new rule were congruent (Figures 3H,I). It should be noted however, that—except for never-reinforced errors—there were no significant genotype-drug interactions (Figures 3B–I), so that a *selective* efficacy of NAC in KO, as opposed to WT mice, cannot be strictly concluded. From an intention-to-treat point-of-view, however, an improved cognitive flexibility in KO-mice, as such, is evident.

### Chronic N-acetylcysteine treatment reduces anhedonia selectively in cyclin-D2 knockout mice

Finally, we assessed anhedonia, a deficit in the negative domain, which we found previously in CD2-KO mice (Grimm et al., 2018). Before sucrose-preference testing (Figure 3J) started, we confirmed that neither group had altered basic drinking volume (*p* > 0.1 for effects of drug, genotype, and drug-genotype interaction, univariate ANOVA; Figure 3K). While mice of all groups displayed a preference for sucrose-containing over normal drinking water, there was a significant drug-genotype interaction (*p* = 0.042, univariate ANOVA) which was driven by two effects: (i) a reduced sucrose-preference in untreated CD2-KO compared to wild-type mice confirming the anhedonia phenotype of this mouse model, and (ii) an increased sucrose-preference in NAC-treated compared to untreated CD2-KO mice (*p* < 0.05, Šidák *post-hoc* tests; Figure 3L). This result demonstrates a full and selective rescue of the anhedonia-phenotype of CD2-KO mice by pre-treatment with NAC.

## Discussion

We here demonstrate that chronic treatment with N-acetylcysteine (NAC, 0.9 g/l in drinking water) may improve certain murine correlates of the cognitive and negative symptom domains of schizophrenia in the CD2-KO mouse model that replicates neurophysiological and psychological pathologies of the prodromal stage of this disease. We found a mild improvement of alternation-based working memory that was not specific to the KO-group of the cohort and occurred only with concomitant NAC-treatment, not *after* chronic pre-treatment. Furthermore, rule-shift learning, a form of cognitive flexibility, was improved by chronic NAC-treatment in the CD2-KO group due to a lower rate of perseverative and random errors, compared to untreated knockouts. Finally, the anhedonia phenotype of CD2-KO mice was selectively rescued by NAC pre-treatment. In contrast, excessive novelty-induced hyperlocomotion, a murine correlate of the positive symptom domain, was neither ameliorated *during* nor *after* chronic NAC treatment. We cannot currently determine, if the treatment during juvenile age (∼P22–P70) would have been sufficient to cause the beneficial effects on cognitive flexibility and anhedonia, or if they were induced by the second treatment during adulthood. While this remains to be assessed in a future study, it has been established in rat models of depression that sub-chronic pre-treatment in adulthood *alone* is sufficient to improve sucrose preference (Fernandes and Gupta, 2019; Chakraborty et al., 2020a,b).

Our findings align with the observation of beneficial effects of acute and long-term NAC-treatment on deficits in the cognitive and negative symptom domains found with rodent models of schizophrenia, depression, or related neuropsychiatric conditions (summarized in Table 1; Schiavone and Trabace, 2018). Most relevant to our current results, long-term NAC treatment was previously found to rescue deficits of cognitive flexibility (Phensy et al., 2017b; Maas et al., 2021) and alternation-based working memory (Phensy et al., 2017b; Monte et al., 2020) in rodent models of schizophrenia. Alongside beneficial effects on anhedonia (see above), improvements of social interaction found in rodent models of both schizophrenia and depression after chronic or acute NAC treatment (see Table 1) further support its efficacy in the negative symptom domain. Our findings of null effects on novelty-induced hyperlocomotion do, however, contrast with NAC-induced reductions of hyperactivity seen in other models of schizophrenia (Fukami et al., 2004; Monte et al., 2020; Lopes-Rocha et al., 2022). This might relate to the fact that NAC may also *increase* exploratory drive in models of depression (Mahmoodzadeh et al., 2021); since the CD2-KO mouse shows features associated with depression, e.g., a lack of neurogenesis (Jaholkowski et al., 2009), the anti-depressant and the hyperactivity-reducing effects of NAC may cancel each other out in this model. Notably, the inefficacy of NAC on hyperlocomotion in our model also suggests that NAC-treatment did not lead to sufficient engagement of mGluR2/3 receptors—a proposed mechanism of NAC action (Baker et al., 2008)—given that their direct activation may selectively correct novelty-induced hyperactivity in CD2-KO mice (Grimm et al., 2018).

Against this backdrop of pre-clinical data, our results further support the notion that chronic NAC treatment may be effective in reducing negative and cognitive symptoms in both prodromal at-risk individuals and patients with schizophrenia. While the current data does not allow to conclude a *specific* efficacy in the prodrome by a NAC-based early intervention nor its capacity to *halt* disease progression, it suggests that clinical trials in at-risk individuals are warranted (Bradlow et al., 2022); indeed, one such multi-center trial is currently conducted (Schmidt et al., 2019). Our results may justify such early-intervention trials based on the prospect of improving negative and cognitive symptoms that are already present in prodromal patients (McGorry, 2011; Fusar-Poli et al., 2013; Miyake et al., 2016), whereas the conduct of such trials could then reveal further beneficial effects on schizophrenia onset in a secondary, longitudinal analysis.

## Data availability statement

The raw data supporting the conclusions of this article will be made available by the authors, without undue reservation.

## Ethics statement

The animal study was reviewed and approved by Regierungspräsidium Tübingen.

## Author contributions

LB and DK conducted the behavioral experiments and analyzed the resulting data. SK provided the task scripts and support for operant testing. DK designed the study. LB and DK wrote the manuscript which was revised and approved by all authors.
